# Use of Epidemic Intelligence from Open Sources for global event-based surveillance of infectious diseases for the Tokyo 2020 Olympic and Paralympic Games

**DOI:** 10.5365/wpsar.2022.13.3.959

**Published:** 2019-09-15

**Authors:** Manami Yanagawa, John Carlo Lorenzo, Munehisa Fukusumi, Tomoe Shimada, Ayu Kasamatsu, Masayuki Ota, Manami Nakashita, Miho Kobayashi, Takuya Yamagishi, Anita Samuel, Tomohiko Ukai, Katsuki Kurosawa, Miho Urakawa, Kensuke Takahashi, Keiko Tsukada, Akane Futami, Hideya Inoue, Shun Omori, Hiroko Komiya, Takahisa Shimada, Sakiko Tabata, Yuichiro Yahata, Hajime Kamiya, Tomimasa Sunagawa, Tomoya Saito, Viema Biaukula, Tatiana Metcalf, Dina Saulo, Tamano Matsui, Babatunde Olowokure

**Affiliations:** a World Health Organization Regional Office for the Western Pacific, Manila, Philippines.; b National Institute of Infectious Diseases, Tokyo, Japan.; * These authors contributed equally.

The Tokyo 2020 Olympic and Paralympic Games (the Games) were postponed for a year due to the coronavirus disease (COVID-19) pandemic. They were finally held from late July to early September 2021. Approximately 83 000 athletes, staff, press and sponsors from over 200 countries and areas attended the event and were hosted across Japan’s  47 prefectures.

Mass gatherings can pose a risk of public health emergencies, and event-based surveillance (EBS) for these events is highly recommended. (*1*) EBS is the organized collection and triage of public health signals that are systematically verified and assessed based on their risk to public health. (*2*) It is used to detect public health signals in countries where mass gatherings occur, as well as public health threats from participating countries. (*1*) During the London 2012 Olympic and Paralympic Games, the Health Protection Agency (currently Public Health England) implemented EBS to provide timely and reliable national epidemic intelligence. EBS sourced events by screening local health authority reports and electronic applications. (*3*)

Public health and social measures were in place to respond to COVID-19 during the Games. However, the threat of importation of non-COVID-19 infectious diseases and their subsequent spread in the community remained. Early detection of acute public health events occurring outside of Japan could have triggered the early response and mitigation of these public health incidents occurring during the Games.

The Japanese National Institute of Infectious Diseases (NIID) conducted enhanced EBS to capture infectious diseases occurring overseas during the Games, (*1*) which comprised their pre-existing EBS system plus external systems. The Epidemic Intelligence from Open Sources (EIOS) system, operated by the World Health Organization (WHO) Regional Office for the Western Pacific, was one of the external systems used. EIOS was built to assist in the early detection, verification, assessment and communication of public health signals and events (*4*) by capturing and aggregating publicly available information, categorizing the information with keywords and providing the results in a secure dashboard. EIOS enables users to monitor media articles of interest on the dashboard by filtering pre-identified keywords, such as the names of countries and diseases. (*5*) EIOS was the main surveillance tool used for the Games to capture articles on infectious diseases and other public health threats occurring outside of Japan.

We describe the experiences and lessons learned from using EIOS for enhanced EBS and risk assessment during the Games. We focused on the screened and assessed media articles on infectious diseases, the continued improvement of artificial intelligence in advancing the use of EIOS as a surveillance tool in mass-gathering events, and collaboration and information sharing between NIID and the WHO Regional Office.

## Methods

### Design and planning

**Fig. 1:**
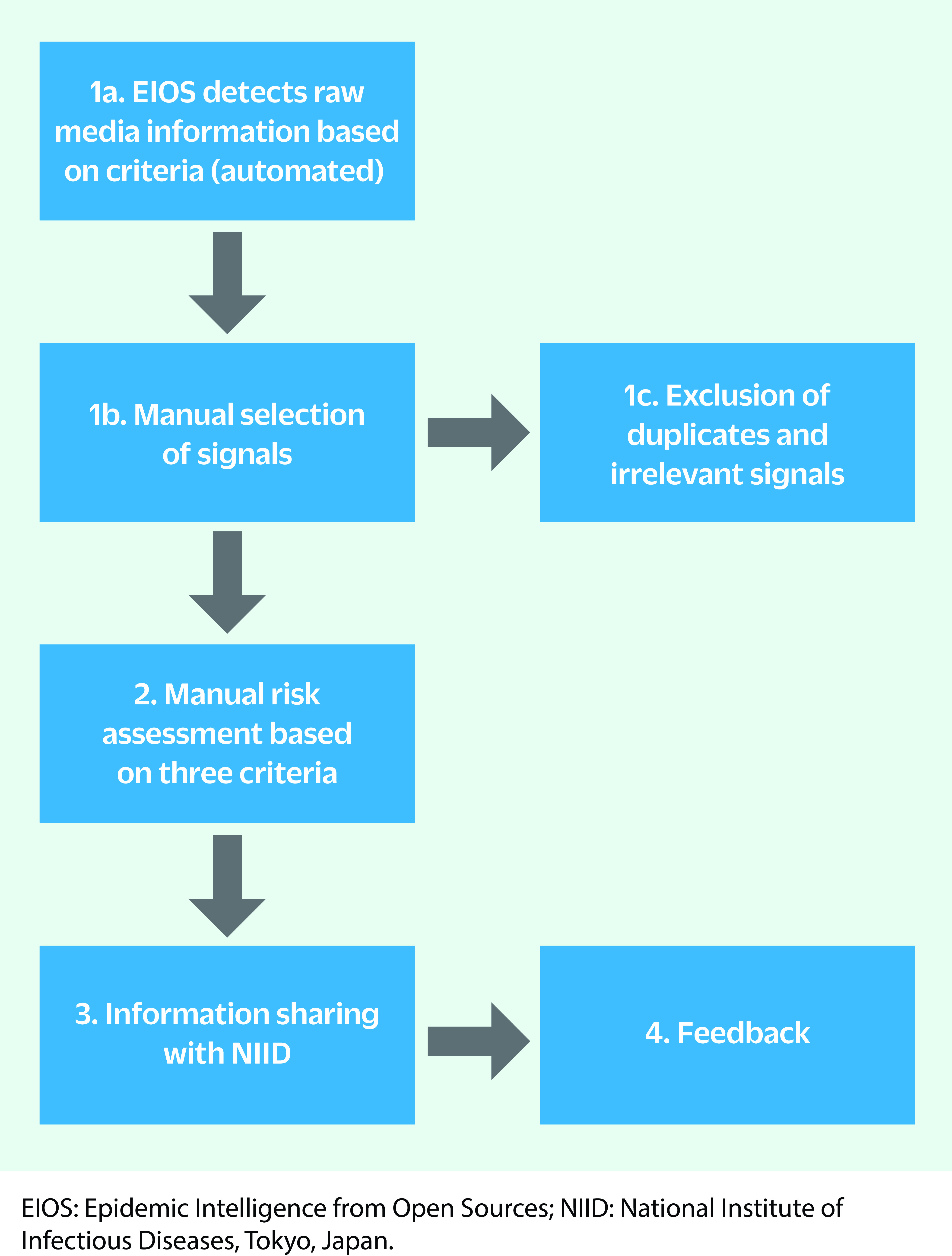
Flow chart of EIOS use during the Tokyo 2020 Olympic and Paralympic Games for event-based surveillance and risk assessment

The planning of routine and ad hoc surveillance activities, as well as the information-sharing mechanisms included in the enhanced EBS using EIOS (**Fig. 1**), were jointly determined by NIID and the WHO Regional Office before the start of EBS operations. Enhanced EBS and risk assessment for the Games was conducted from 1 July to 19 September 2021, covering the period before and after both the Olympic and Paralympic Games, which were held from 23 July to 8 August 2021 and from 24 August to 5 September 2021, respectively.

[**insert** Figure 1]

### Data collection using Epidemic Intelligence from Open Sources

**Fig. 2:**
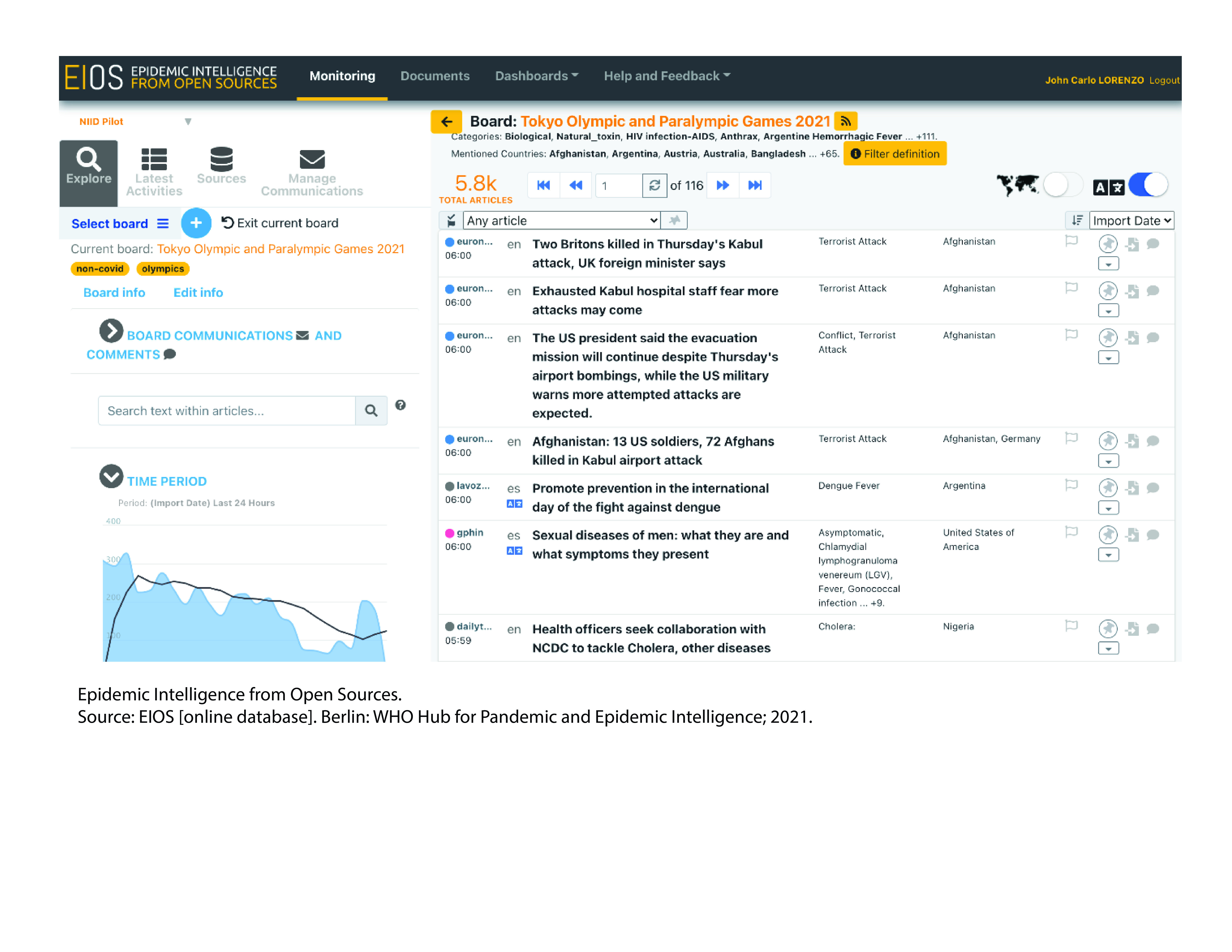
Example of how the EIOS dashboard was used for the Tokyo 2020 Olympic and Paralympic Games

EIOS was identified as a suitable tool to use for screening publicly available online media articles and sources for unverified reports referencing infectious diseases. With support from the Information Systems and Data Management Team at WHO headquarters, the Tokyo 2020 EIOS dashboard was developed by late June 2021 using the agreed sets of countries, infectious diseases and other public health threats to be screened using EIOS (**Fig. 2**). The selection of 69 countries and areas (**Box 1**) from Africa, the Americas, Asia, Europe and Oceania was made based on the number of participants and delegations to the two previously held Games. (*1*) Further, the selection of infectious diseases of interest (**Box 2**) was determined by the prevalence of these diseases among the selected countries. Signals about the risk of bioterrorism and outbreaks of unknown origin were also captured.

[**insert** Figure 2]

Box 1 **Initial EIOS criteria for screening targeted countries and areas during the Tokyo 2020 Olympic and Paralympic Games**

- Africa: Egypt, Kenya, Morocco, Nigeria, South Africa, Tunisia.
- Americas: Argentina, Brazil, Canada, Colombia, Cuba, Jamaica, Mexico, Peru, United States of America, Venezuela.
- Asia: Afghanistan, Bangladesh, Cambodia, China, Hong Kong Special Administrative Region SAR (China), India, Indonesia, Islamic Republic of Iran, Kazakhstan, Malaysia, Mongolia, Myanmar, Nepal, Pakistan, Philippines, Republic of Korea, Singapore, Sri Lanka, China, Taiwan (China), Thailand, Uzbekistan, Viet Nam.
- Europe: Austria, Belarus, Belgium, Bulgaria, Croatia, Czech Republic, Denmark, Finland, France, Germany, Greece, Hungary, Ireland, Italy, Lithuania, Netherlands, Norway, Poland, Portugal, Romania, Russian Federation, Serbia, Slovenia, Spain, Sweden, Switzerland, Turkey, Ukraine, United Kingdom of Great Britain and Northern Ireland.
- Oceania: Australia, New Zealand.

Source: EIOS [online database]. Berlin: WHO Hub for Pandemic and Epidemic Intelligence; 2021.

Box 2 **Initial EIOS criteria for screening targeted infectious diseases and events during the Tokyo 2020 Olympic and Paralympic Games**

- Human-to-human: acute gastroenteritis, bacterial meningitis, diphtheria, hepatitis B, influenza, measles, meningococcal infection, Middle East respiratory syndrome, mumps, pertussis, polio, rubella, sexually transmitted infections (chlamydia infection, gonococcal infection, HIV, syphilis), tuberculosis, varicella.
- **Foodborne: amoebiasis, botulism, cholera, cryptosporidiosis, enterohaemorrhagic** *Escherichia coli*, **giardiasis, hepatitis A, hepatitis E, listeriosis, shigellosis, typhoid/paratyphoid.**
- **Soil/waterborne: coccidiosis,** *Cryptococcus gattii* **infection, histoplasmosis legionellosis, leptospirosis, melioidosis, strongyloidiasis, tetanus.**
- Zoonosis: anthrax, avian influenza, brucellosis, hantavirus infection, Hendra virus infection, Lassa fever, monkeypox, Q fever, rabies, Rift Valley fever, Rissa virus infection, South American haemorrhagic fever, tularaemia.
- Mosquito-borne: Barmah Forest virus infection, chikungunya, dengue, East equine encephalitis, Japanese encephalitis, La Crosse encephalitis, malaria, Oropouche fever, Ross River virus infection, Saint Louis encephalitis, West equine encephalitis, West Nile fever, yellow fever, Zika virus disease.
- Tick-borne: African spotted fever, anaplasmosis, Crimean-Congo haemorrhagic fever, Colorado tick fever, ehrlichiosis, Kyasanur Forest fever, Lyme disease, Omsk haemorrhagic fever, Powassan encephalitis, Queensland tick typhus, recurrent fever, severe fever with thrombocytopenia syndrome, spotted fever (Mediterranean spotted fever, Rocky Mountain spotted fever and other spotted fever groups), tick-borne encephalitis.
- Other arthropod-borne: Chagas disease, leishmaniasis, plague, scrub typhus.
- Potential risk of bioterrorism: white powder, attack.
- Disease outbreaks with unknown etiology: symptoms (coma, respiratory, diarrhoea, haemorrhage, fever).

Source: EIOS [online database]. Berlin: WHO Hub for Pandemic and Epidemic Intelligence; 2021.

### Data collection process

An automated exclusion process was conducted by EIOS to filter out the diseases and countries not included in the pre-identified categories of countries and infectious diseases. During manual screening by a WHO Regional Office staff member, duplicates and irrelevant articles were discarded. For screened media articles requiring further verification, epidemiological data on the infectious disease of interest were collected manually from the reporting country. Media articles that were considered to indicate public health risks were regarded as signals and were then compiled in a daily media screening report. This report includes the category of the disease of interest in each media signal, a summary of the available information  on the situation, and the continent and country where the signal was reported. When available, details on the action and response taken by the local health authorities were included to support the risk assessment.

### Risk assessment

Each selected media signal was assessed using the following criteria:

Criterion 1: Does the condition have the likelihood of importation of infectious disease? (Yes/No)
Criterion 2: Does the condition have the likelihood of transmission among Games personnel and the community? (Yes/No)
Criterion 3: Does the condition have the likelihood of having a significant impact on society? (Yes/No)

If criterion 1 was marked “No,” criteria 2 and 3 were not assessed. Criterion 3 focused on bioterrorism signals as they can have a significant impact on society. Additional information on the disease, including seasonality, trends, recent outbreaks and other epidemiological data, were collected and shared with NIID to increase confidence in the assessment for each criterion.

### Information sharing and feedback

The assessed signals compiled in the daily media screening reports by the WHO Regional Office were shared with NIID on a daily basis for their assessment against the Playbooks, which were a set of guidelines prepared by the Tokyo Organizing Committee of the Olympic and Paralympic Games that outlined the responsibilities and rules of all the Games participants and Games-related personnel. They were also compiled by NIID in the daily situational report, together with data on priority notifiable infectious diseases in Japan and COVID-19 information relevant to the Games. The daily situational report was disseminated to Japan’s local health authorities and to WHO through the International Health Regulations (IHR) communication mechanism.

## Results

Between 1 July and 19 September 2021, a total of 103 830 media articles appeared on the Tokyo 2020 EIOS dashboard. Of these, 5441 (5.2%) were deemed relevant to public health threats and manually screened, out of which 587 (0.6%) were regarded as signals and were reported to NIID (**Table 1**).

**Table 1:** Number and proportion of signals detected through the EIOS dashboard for the Tokyo 2020 Olympic and Paralympic Games, assessment outcomes and reported diseases that met criteria 1 and 2, 1 July to 19 September 2021

Signals	Number of articles (%)
Detected through EIOS (*n* = 103 830)	
Not screened (did not meet selection criteria)	98 389 (94.7)
Screened and discarded	4854 (4.7)
Screened and reported as signals	587 (0.6)
Assessment of signals (*n* = 587)	-
“No” for criterion 1^a^	329 (56.0)
“Yes” for criterion 1	258 (44.0)
“Yes” for criteria 1 and 2^b^	211 (35.9)
“Yes” for criterion 3^c^	2 (0.3)
Reported diseases of signals that met criteria 1 and 2 (*n* = 211)	
Mosquito-borne diseases	173 (82.0)
Sexually transmitted infections	29 (13.8)
Unknown diseases	6 (2.8)
Others	3 (1.4)

EIOS: Epidemic Intelligence from Open Sources.

^a^ Criterion 1: Does the condition have the likelihood of importation of infectious disease?

^b^ Criterion 2: Does the condition have the likelihood of transmission among Tokyo 2020 personnel and the community?

^c^ Criterion 3: Does the condition have the likelihood of having a significant impact on society?

Among the 587 signals, 211 (35.9%) had “Yes” for both criteria 1 and 2, emphasizing the likelihood of their importation into Japan through the Games and spread to the local community. About 82% (173 of 211 with “Yes” for criteria 1 and 2) were mosquito-borne diseases such as dengue, chikungunya and Zika virus disease. Of these  173 mosquito-borne disease signals, dengue accounted for 139 (80.3%). The WHO South-East Asia Region and the WHO Region of the Americas reported the most dengue signals with 78 (56.1%) and 39 (28.1%) signals, respectively.

Sexually transmitted infections were the next most common at 13.7% (29/211), and diseases with unspecified causative agents accounted for the remaining 2.8% (6/211) of signals. Of all reported signals, 0.3% (2/587) had “Yes” for criterion 3, implicating the likelihood of having a significant impact on society.

None of the signals detected were assessed as having the likelihood of a significant impact on the Games. Further, none of the signals required the activation of the IHR communication mechanism.

## Discussion

EIOS provided an enhanced surveillance system with quality-assured risk assessment for the Games. None of the 587 signals reported had a potentially significant impact on the Games. One of the possible reasons may be the significant decrease in infectious disease activity due to public health and social measures for COVID-19 globally. Population mobility restrictions, international and domestic travel measures, and school closures resulted in the decline of several infectious diseases, especially vaccine-preventable diseases. (*6*-*8*) Decreases were also observed for respiratory infectious diseases globally, during and after the implementation of community control strategies for COVID-19. (*9*-*11*) However, some decrease in cases of infectious diseases might be caused by potential underdetection due to less opportunity for testing and/or delays in final diagnosis as a consequence of overwhelmed health-care systems and the fear of being treated as a suspected COVID-19 case. (*12*, *13*) Even though none of the detected signals were considered significant, the detection, monitoring and information-sharing processes pertaining to acute public health events occurring outside Japan were valuable.

As EIOS displays publicly available articles from multiple sources tagged by pre-identified categories, it was considered a good tool to capture information on infectious diseases occurring globally. However, EIOS displays multiple replicated articles, revealing duplication of effort in conducting EBS screening activities. Due to its sensitivity, EIOS also displays irrelevant articles which significantly increases the number of articles tagged for events with high media attention.

So as to improve the use of EIOS as a mass gathering surveillance tool, continued use and improvement of artificial intelligence that selects and clusters articles with duplicate content before being displayed on the EIOS dashboard should be considered. Clustering similar media signals would lessen the time spent manually screening the results as duplicated content would only appear once. It would also show if a signal has high media attention without omitting valuable information from other media articles. Moreover, inclusion and exclusion features of a specific category based on international political and social conditions would be effective in reducing irrelevant articles and minimizing the clamour from incidents with high international media attention. An additional function able to search articles from an official information source may also contribute to increasing specificity and reducing the time spent manually screening EIOS articles.

The major advantage of using EIOS during the Games was the timely and consistent identification of global epidemiological information, which complemented NIID’s other EBS activities and supported the conduct of appropriate risk assessment. (*1*) This timely detection and quality-assured risk assessment enabled the Japanese Ministry of Health, Labour and Welfare (MHLW) and the WHO Regional Office to consider whether facilitating IHR communication for further verification was necessary. Through collaboration and information sharing, and having EIOS managed externally, MHLW and NIID were able to receive relevant information on potential public health events that could have resulted in imported disease during the Games. EIOS was a successful component of the enhanced surveillance system for infectious diseases and public health threats that could have impacted the Games.
